# Supplementary material for: Immunogenicity and Safety of Childhood Combination Vaccines: A Systematic Review and Meta-Analysis
Source: Vaccines (Basel). 2022 Mar 18;10(3):472. doi: 10.3390/vaccines10030472 (PMC8954135; doi:10.3390/vaccines10030472)
Supplement: Supplementary file 1 [file vaccines-10-00472-s001.zip › Supplementary Table S1 and Table S2.pdf]

Supplementary Table S1. Antigenic content of some of the DTaP-based combined vaccines

			Antigen contents						
**Vaccine**	**Trade name**	**Manufacturer**	D	T	aP	HBV	Hib	IPV	**Age**
DTaP-HBV-IPV	Pediarix	GlaxoSmithKline	√	√	√	√	-	√	6 weeks to 6 years
DTaP-IPV-Hib	Pentacel	Sanofi Pasteur	√	√	√	-	√	√	6 weeks to 4 years
DTaP-HBV-Hib-IPV	Infanrix hexa	GlaxoSmithKline	√	√	√	√	√	√	6 weeks to 2 years

DTaP: Diphtheria, Tetanus, acellular Pertyussis; HBV: Hepatitis B Virus; Hib: Haemophilus influenzae type b; IPV: Inactivated Polio Virus.

Supplementary Table S2. Antigen composition of DTaP-HBV-IPV-Hib

	**DTaP-HBV-Hib-IPV**
Diphtheria toxoid (DT)	DT (>30 IU）absorbed on $Al(OH)_3$
Tetanus toxoid (TT)	TT (>40 IU）adsorbed on $Al(OH)_3$
Pertussis antigens	Pertussis toxoid (25μg), filamentous hemagglutinin(25μg), and pertactin(8μg), absorbed on $Al(OH)_3$
Inactivated poliovirus (IPV)	IPV type 1 (Mahoney strain, 40 D-antigen unit), type 2 (MEF-1 strain, 8D-antigen unit), type 3 (Saukett strain, 32 D-antigen unit)
Hacmophilus influenzac typeb (Hib)	Hib polysaccharide (polyribosylribitol phosphate, 10 µg) conjugated to tetanus toxoid (~25 µg), adsorbed on $Al(PO_4)$
Hepatitis B (HepB)	HB surface antigen (10 µg) adsorbed on $Al(PO_4)$

Composition for 1 dose (0.5 mL for each vaccine). IU: international unit; $Al(OH)_3$: aluminum hydroxide, hydrated; $Al(PO_4)$: aluminum phosphate
