# Supplementary figures and images for: Immunogenicity and Safety of Childhood Combination Vaccines: A Systematic Review and Meta-Analysis
Source: Vaccines (Basel). 2022 Mar 18;10(3):472. doi: 10.3390/vaccines10030472 (PMC8954135; doi:10.3390/vaccines10030472)

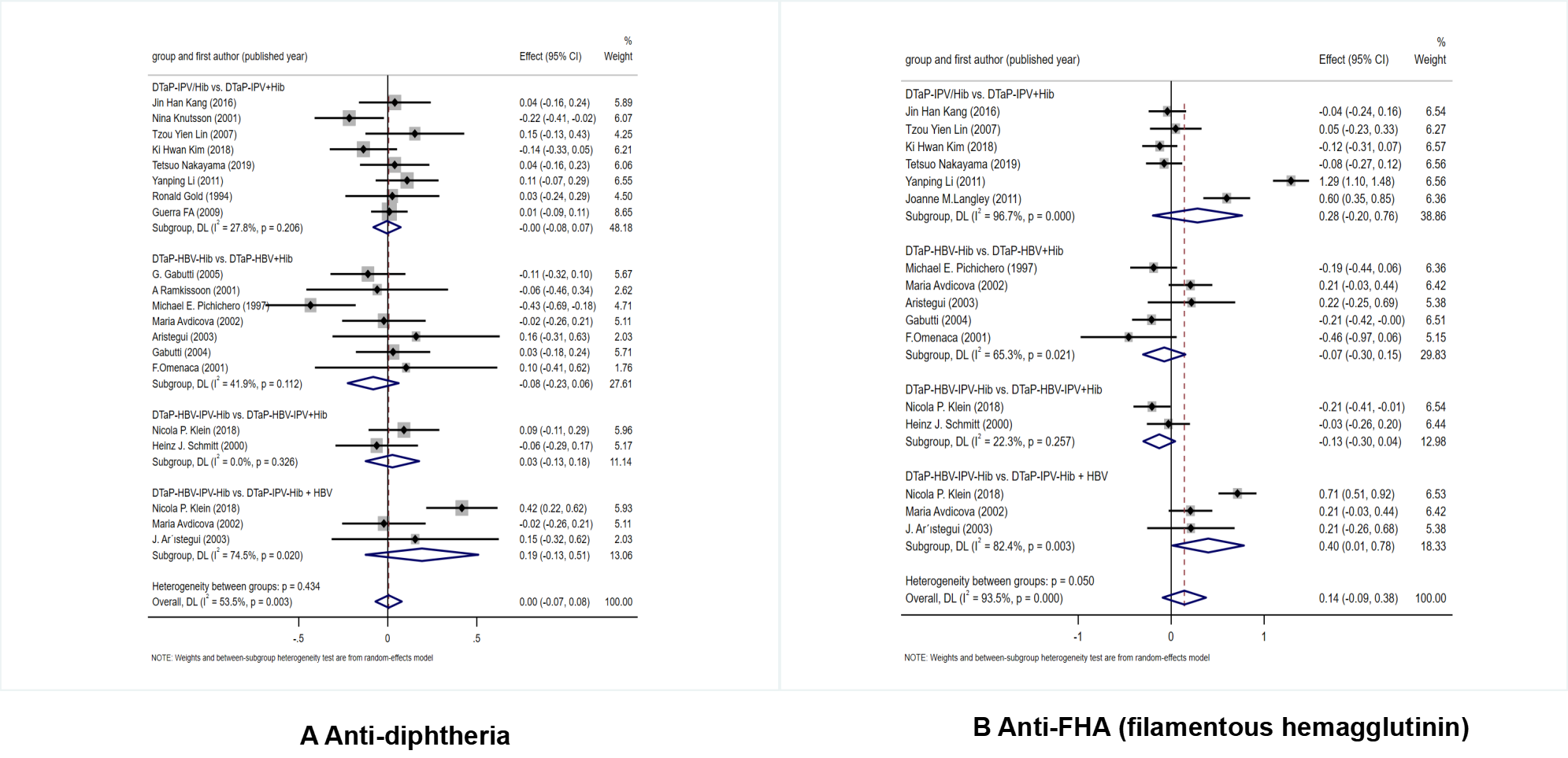

Supplement: Supplementary file 1 [file vaccines-10-00472-s001.zip › Supplementary Figure S1A-B.tif]

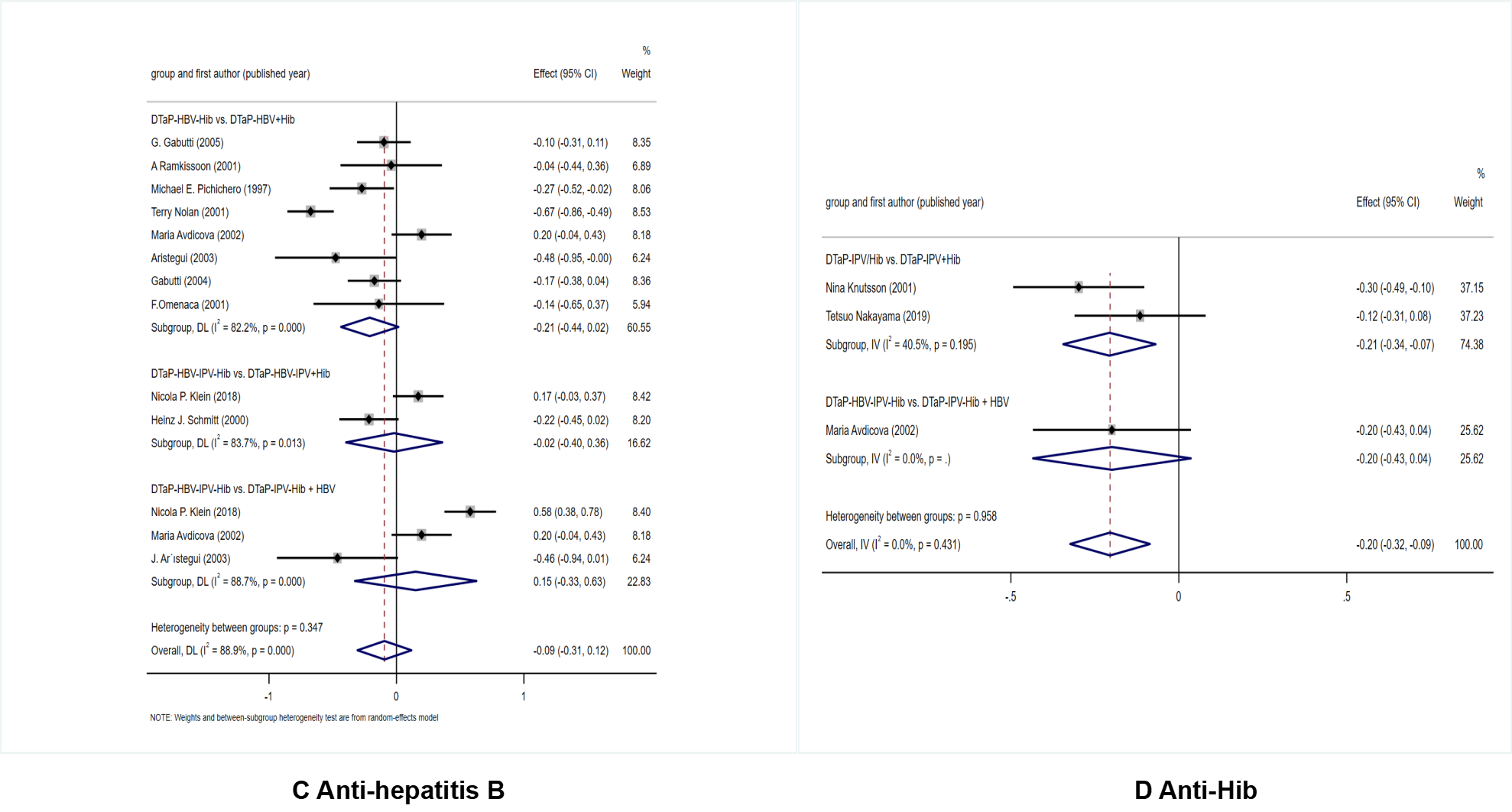

Supplement: Supplementary file 1 [file vaccines-10-00472-s001.zip › Supplementary Figure S1C-D.tif]

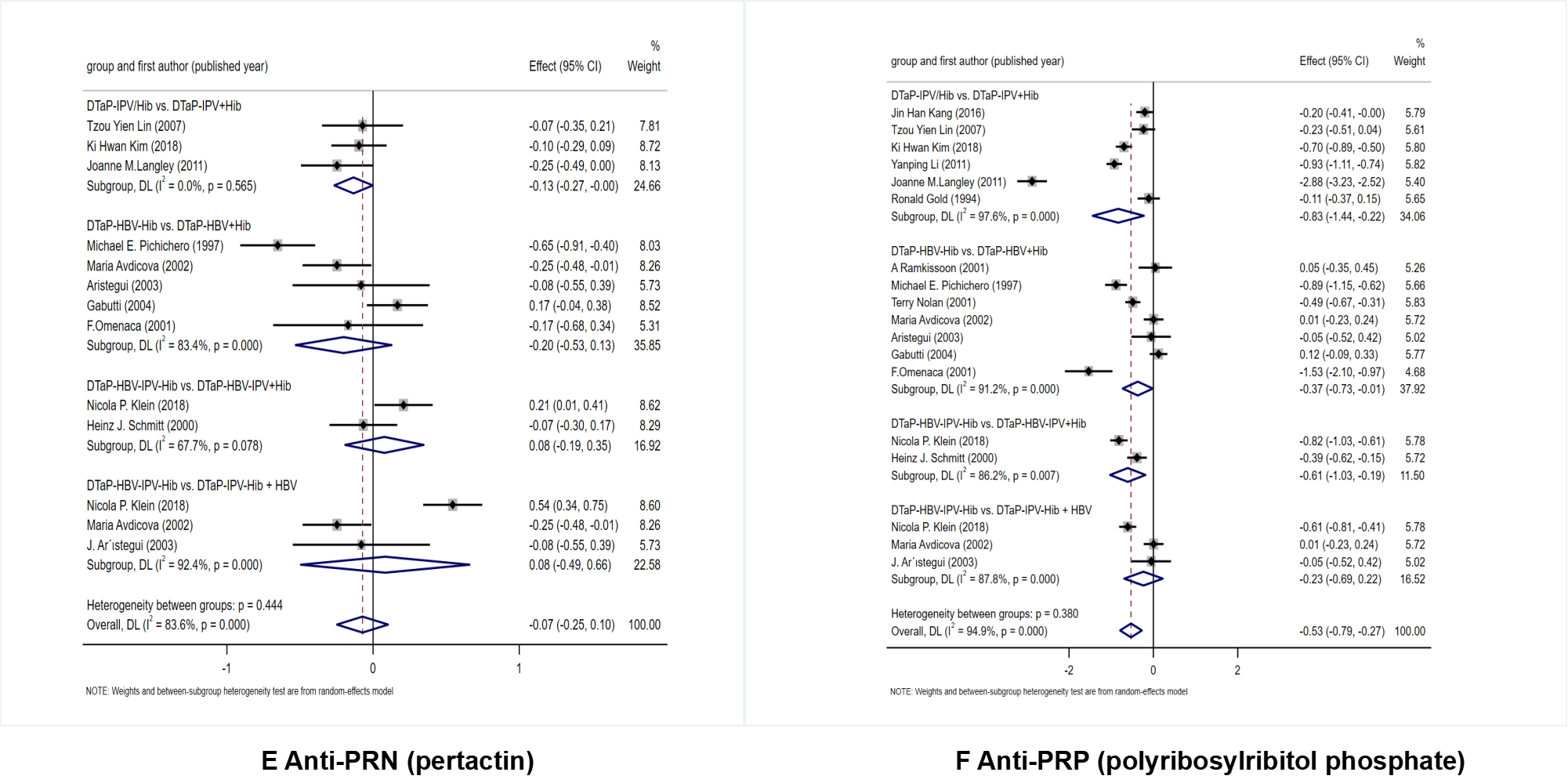

Supplement: Supplementary file 1 [file vaccines-10-00472-s001.zip › Supplementary Figure S1E-F.tif]

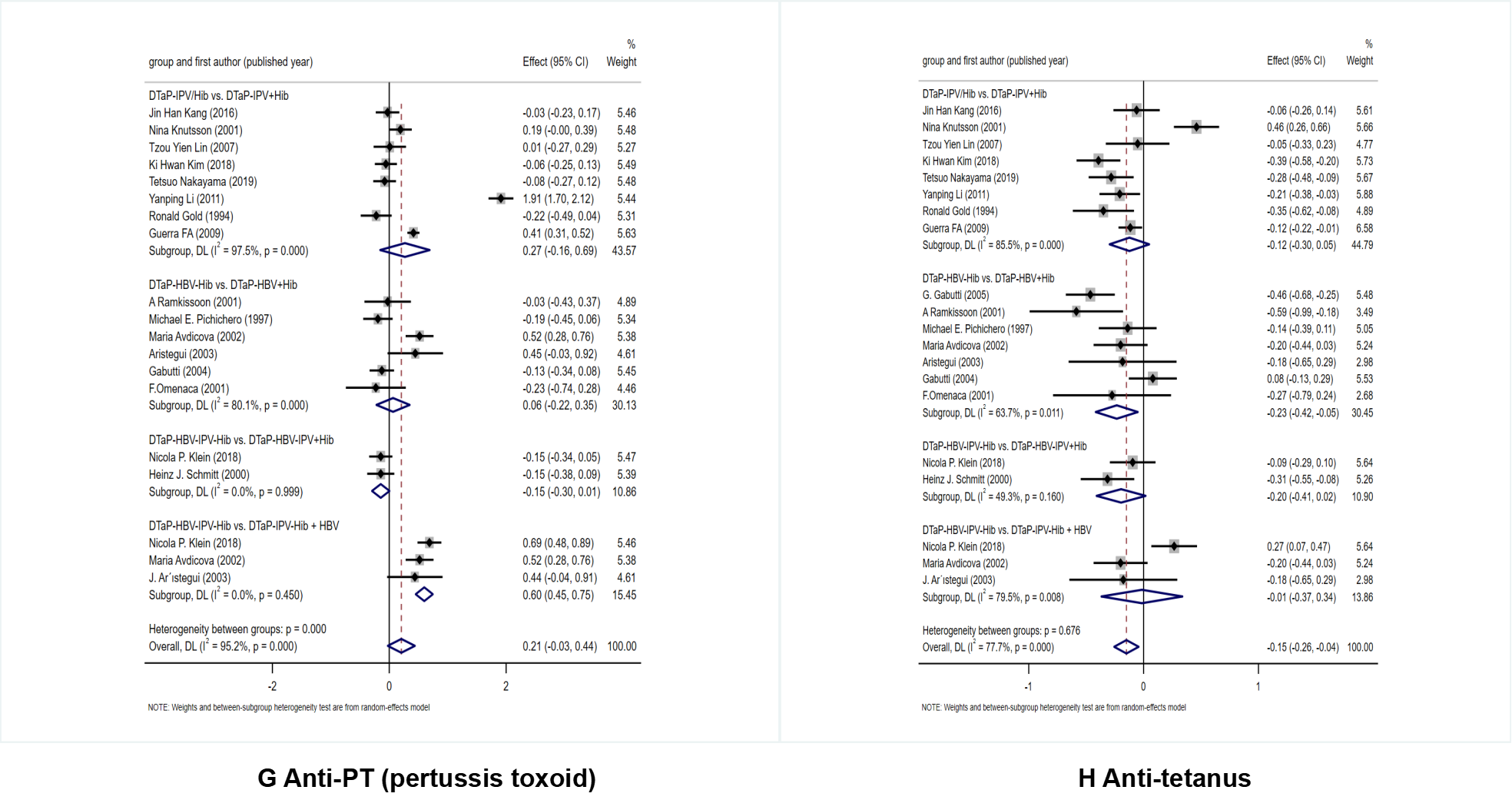

Supplement: Supplementary file 1 [file vaccines-10-00472-s001.zip › Supplementary Figure S1G-H.tif]

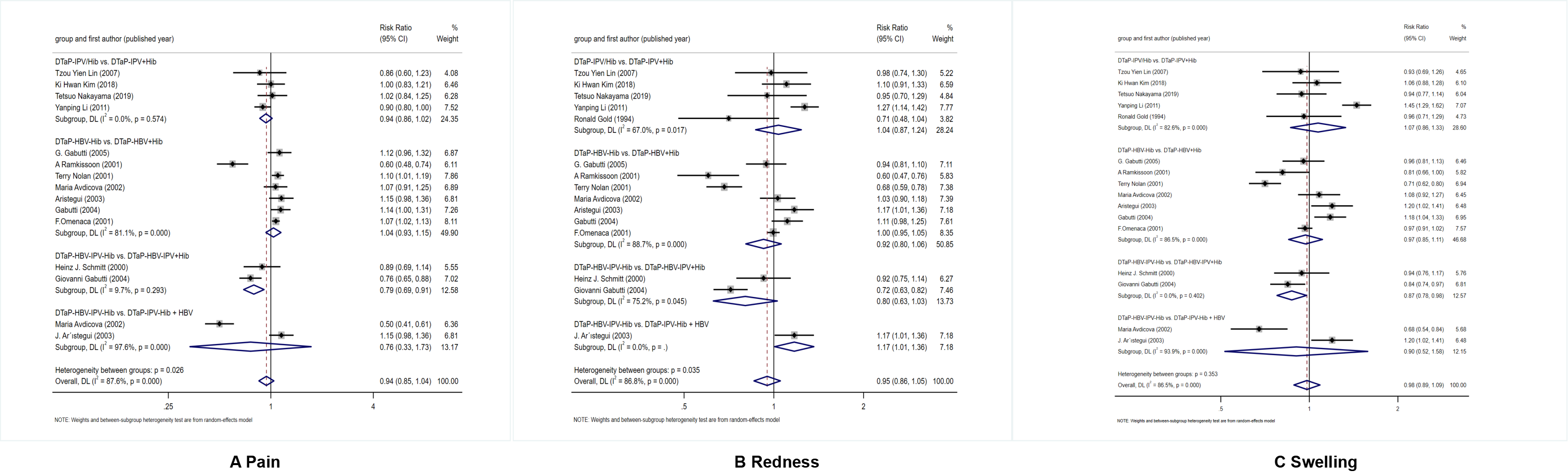

Supplement: Supplementary file 1 [file vaccines-10-00472-s001.zip › Supplementary Figure S2A-C.tif]

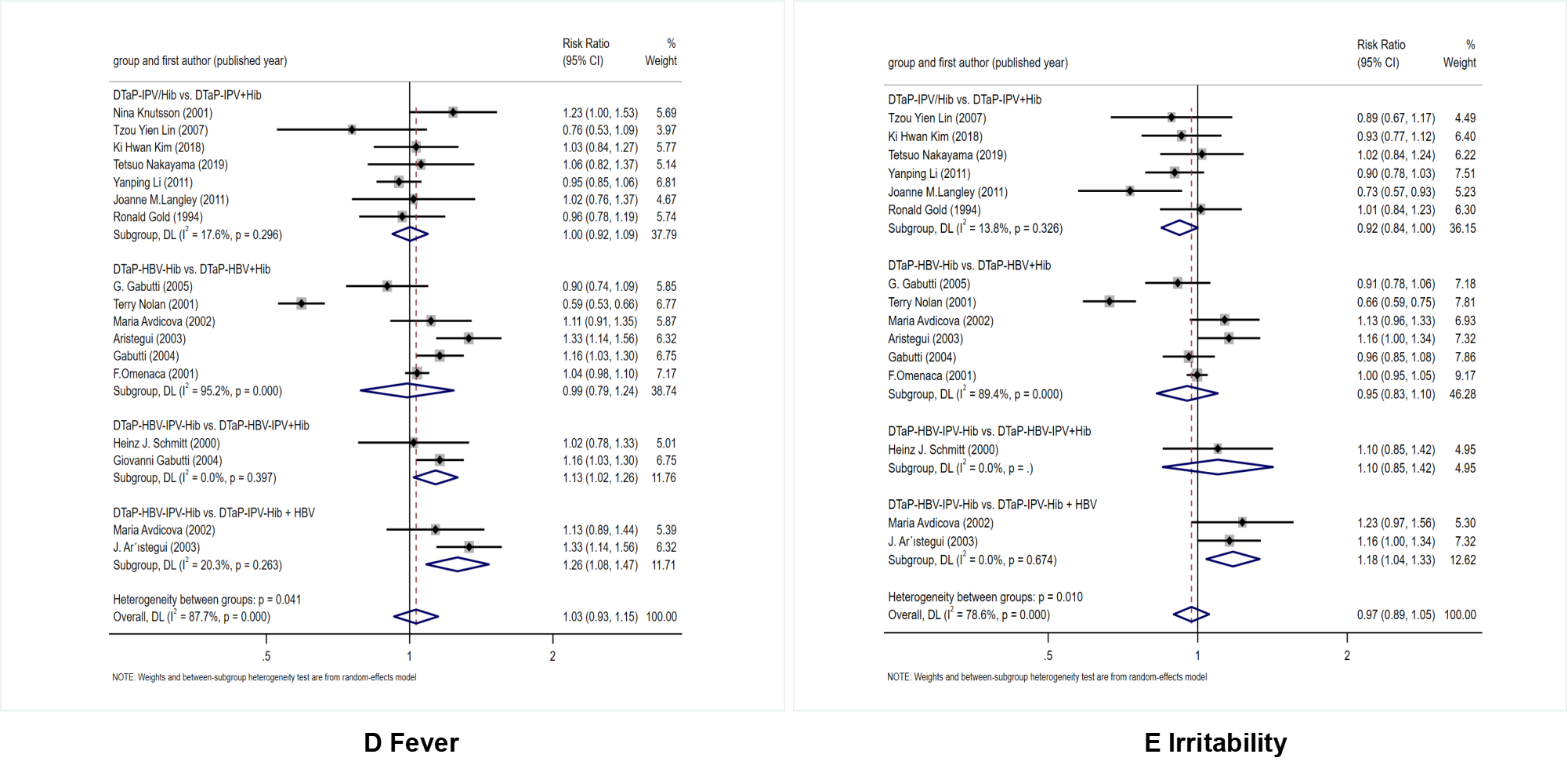

Supplement: Supplementary file 1 [file vaccines-10-00472-s001.zip › Supplementary Figure S2D-E.tif]

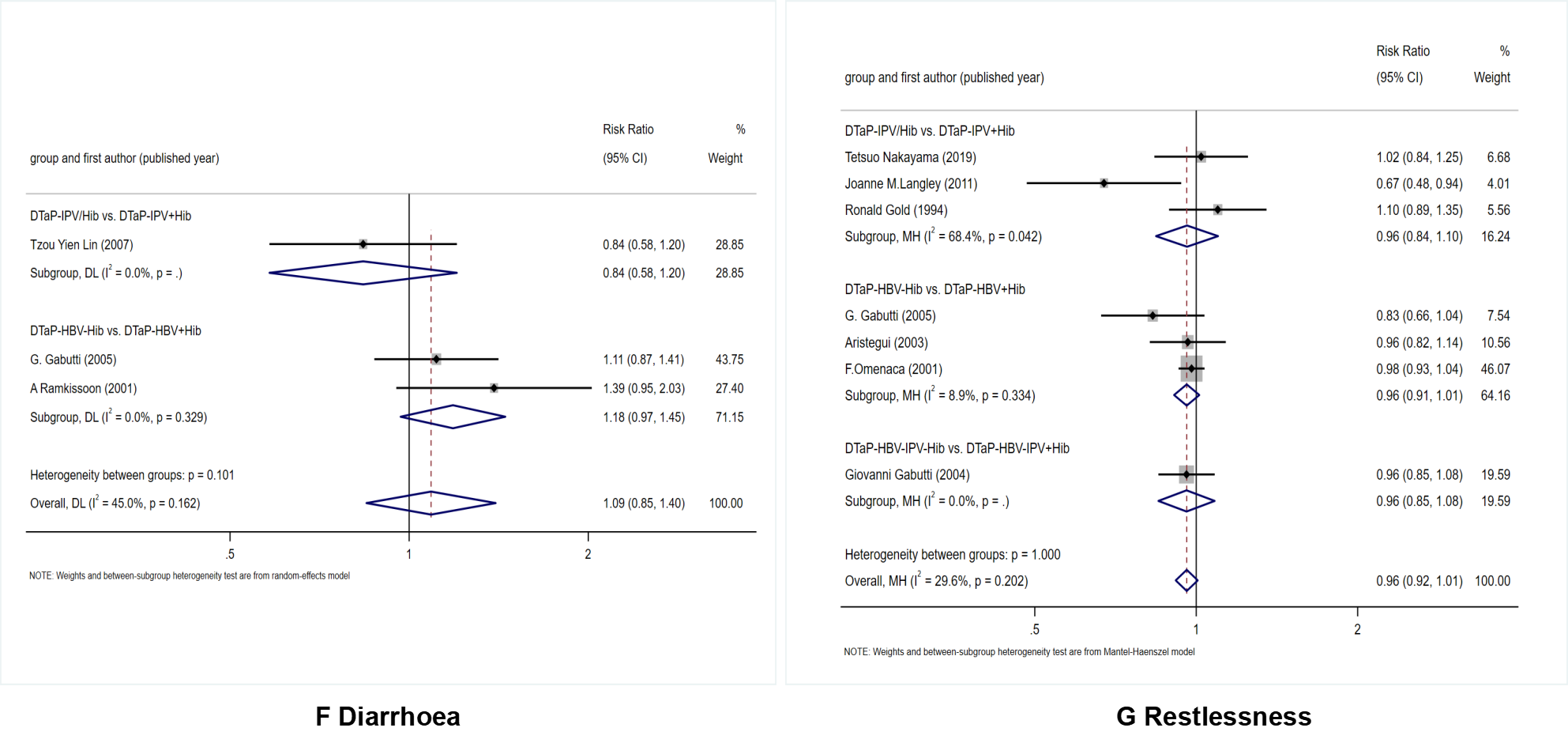

Supplement: Supplementary file 1 [file vaccines-10-00472-s001.zip › Supplementary Figure S2F-G1.tif]

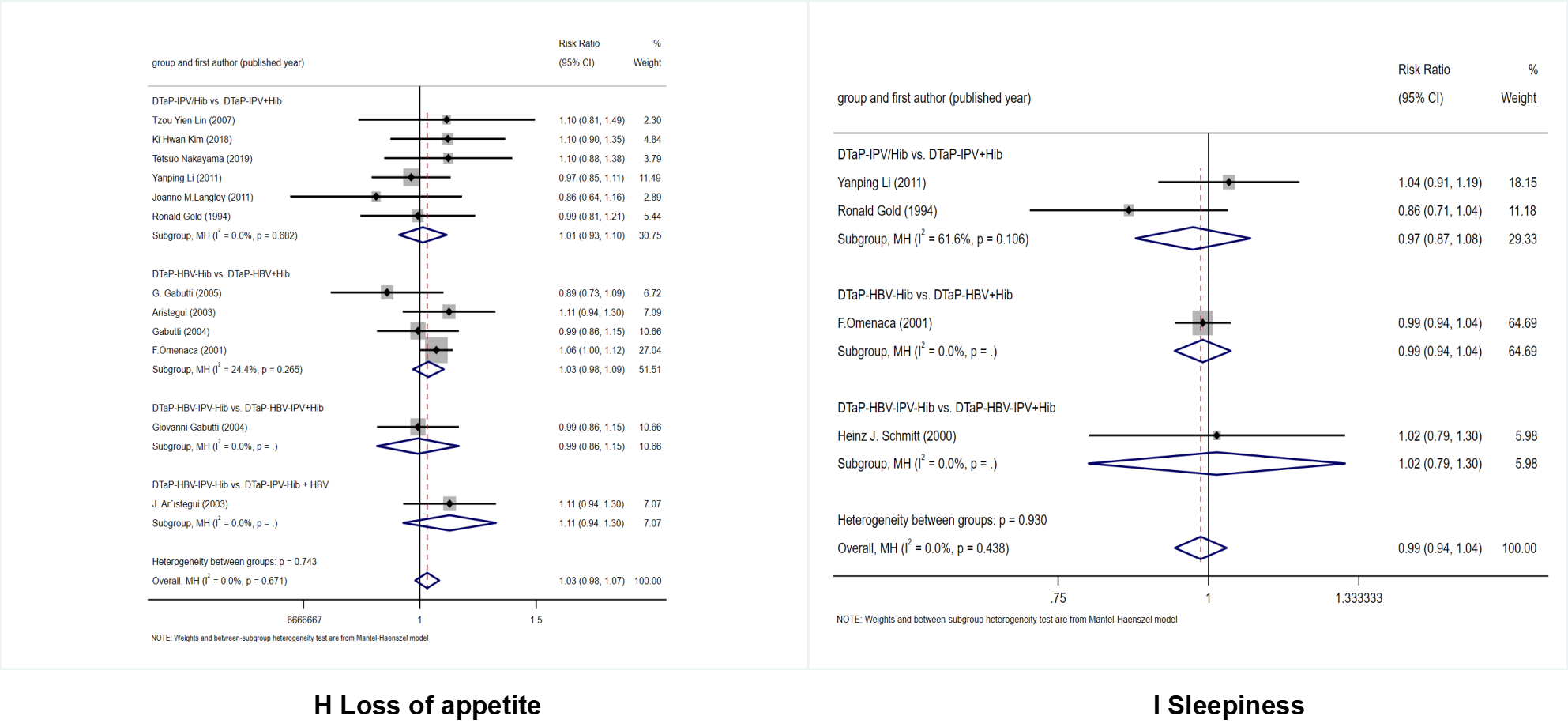

Supplement: Supplementary file 1 [file vaccines-10-00472-s001.zip › Supplementary Figure S2H-I.tif]

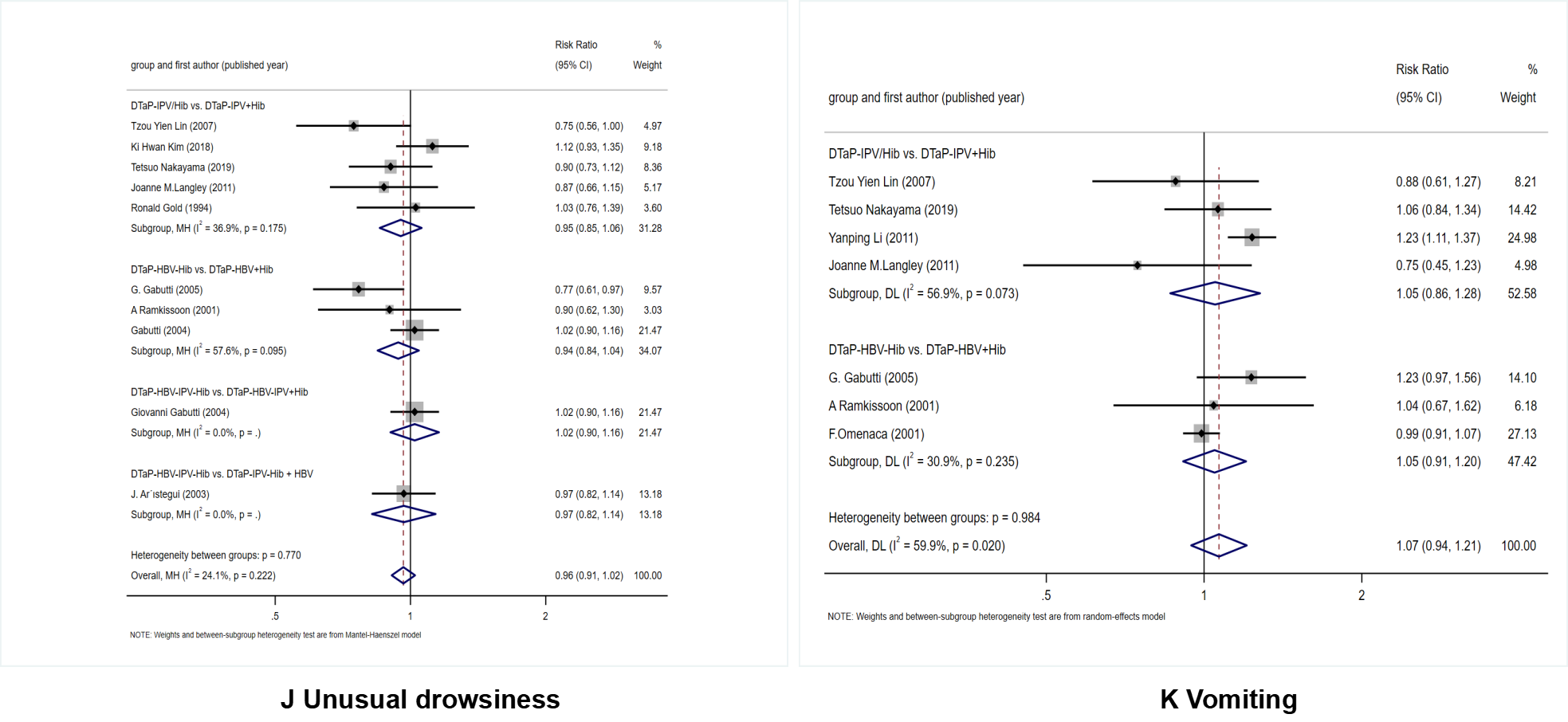

Supplement: Supplementary file 1 [file vaccines-10-00472-s001.zip › Supplementary Figure S2J-K.tif]

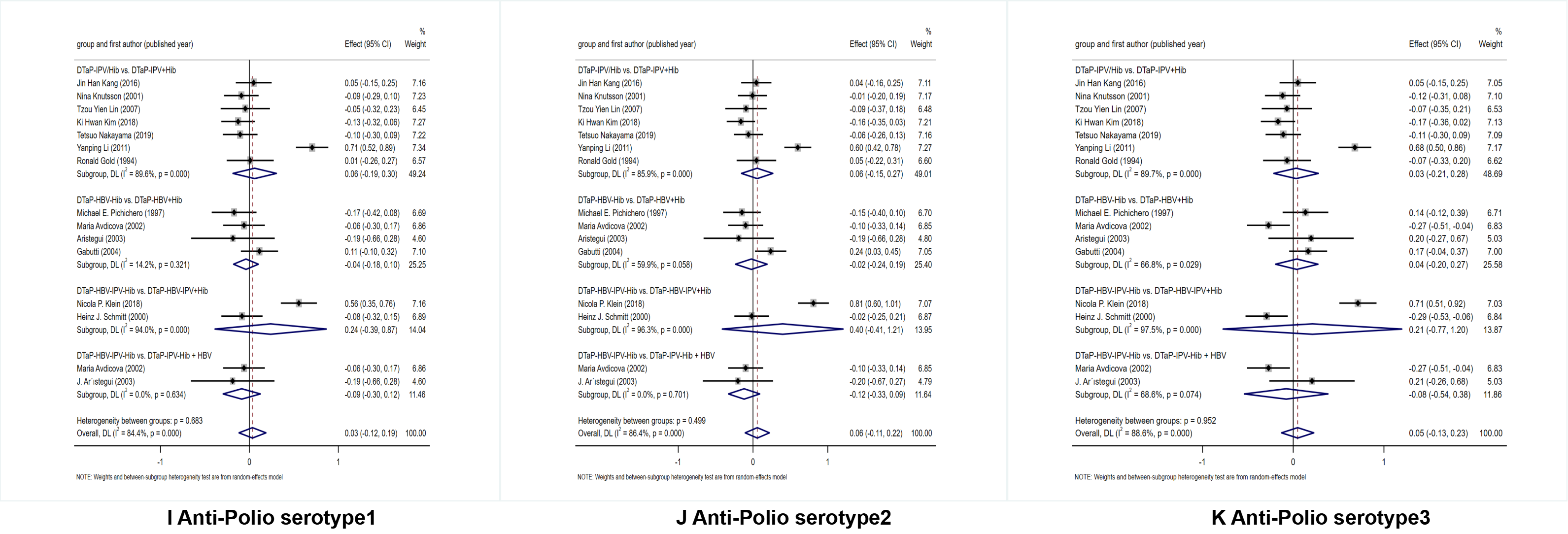

Supplement: Supplementary file 1 [file vaccines-10-00472-s001.zip › Supplementary Figure SI-K.tif]
